# A Smart Wireless Ear-Worn Device for Cardiovascular and Sweat Parameter Monitoring During Physical Exercise: Design and Performance Results

**DOI:** 10.3390/s19071616

**Published:** 2019-04-04

**Authors:** Bruno Gil, Salzitsa Anastasova, Guang Z. Yang

**Affiliations:** The Hamlyn Centre, Imperial College London, London SW7 2AZ, UK; s.anastasova-ivanova@imperial.ac.uk (S.A.); g.z.yang@imperial.ac.uk (G.Z.Y.)

**Keywords:** wearables, ear, electrocardiogram, sweat, lactate, pH, Bluetooth Low Energy

## Abstract

Wearable biomedical technology has gained much support lately as devices have become more affordable to the general public and they can easily interact with mobile phones and other platforms. The feasibility and accuracy of the data generated by these devices so as to replace the standard medical methods in use today is still under scrutiny. In this paper, we present an ear-worn device to measure cardiovascular and sweat parameters during physical exercise. ECG bipolar recordings capture the electric potential around both ears, whereas sweat rate is estimated by the impedance method over one segment of tissue closer to the left ear, complemented by the measurement of the lactate and pH levels using amperiometric and potentiometric sensors, respectively. Together with head acceleration, the acquired data is sent to a mobile phone via BLE, enabling extended periods of signal recording. Results obtained by the device have shown a SNR level of 18 dB for the ECG signal recorded around the ears, a THD value of −20.46 dB for the excitation signal involved in impedance measurements, sweat conductivity of 0.08 S/m at 1 kHz and sensitivities of 50 mV/pH and 0.8 μA/mM for the pH and lactate acquisition channels, respectively. Testing of the device was performed in human subjects during indoors cycling with characteristic level changes.

## 1. Introduction

Wearable biomedical technology has become ubiquitous in recent years, sparking a new research field in the area of IoT for healthcare applications [1]. Electronic miniaturization and advances in smart materials have allowed devices to be attached to the surface of the human body for measurement of physiological parameters, while consuming little amount of power and/or harvesting the energy from the body environment itself. Examples of such devices include glucose meters, biopotential monitors, motion trackers, odor sensors and UV detectors. The requirement for causing minimum discomfort and embarrassment to the user is guiding the design of the so-called *wearables* so as to be conformal with the body shape, in the form of flexible adhesive patches or incorporated into pieces of clothing and accessories. Many of these devices are already on the market in part to satisfy consumers’ appetite for cutting-edge technology, but also to expand the research from the clinical environment to the comfort of home, workplace or sports venue [2]. 

Continuous monitoring of physiological variables is still largely limited to clinical environments and/or specialized laboratories that typically use bulky medical equipment certified for human trials. Such equipment requires galvanically-isolated power and data communication lines for the embedded electronics, as well as wiring connections to the recording spots over the body surface. Measurement of more than one signal modality involves recruiting additional equipment to detect the different signals simultaneously. Therefore, wearable device design must couple, in one end, with the acquisition of signals originated from different transduction mechanisms and, in the other end, employ low-power and low data-rate transfer protocols, like the Bluetooth Low Energy (BLE) for untethered applications that are battery-powered. Much like the case of the present study, where we committed ourselves to measure several signals related with the performance of physical exercise, including the assessment of cardiovascular, motion and sweat parameters, inside a small-form device developed for the head segment. 

Body motion detection has been the target of a large portion of wearable technology developed in the past years, employing innovative ways to integrate accelerometers, gyroscopes and/or magnetometers into different body segments/joints and also taking into account the surrounding environment where the detection is performed [3]. In order to provide motion detection, we opted to include an accelerometer in the proposed device as the information originated by head acceleration can help complement the information derived from cardiovascular and sweat monitoring, by distinguishing if a sudden change in the latter parameters is related to exercise, as targeted by this study, or rather resulting from some pathological condition leading to movement restrictions, such as fever experienced during a systematic inflammatory response of the body. Regarding cardiovascular monitoring, measurement of the electrical activity of the heart has long been the preferential method to estimate heart rate (HR) and heart rate variability (HRV), useful to detect early signs of potential heart attack, arrhythmias and myocardial infection [4,5]. For exercise in particular, control of the individual effort by monitoring HR and HRV more accurately and frequently is important not only to avoid body injuries due to inadequate intensive strains but also to prevent sudden death, as the vast majority of exercises practiced in sport involve a short increase in the risk of heart attack. Modern ECG monitors limit the number of body leads in order to reduce dimensions and power consumption, acquiring the electrical signal preferentially over the chest and/or body limbs. In this study, we opted to acquire the ECG around the ears by using a single bipolar lead, hence preventing any wiring connections over the limbs (and chest) that can disturb exercise’s performance. In the interface with the skin, novel ECG electrodes are also been developed to withstand higher temperatures, humidity levels and water immersion, occurring in some sport modalities. Moreover, special adhesives made of nanomaterials and epidermal electronics have been shown to produce less skin irritation, inasmuch like the E-textiles employed in the present study as an alternative to the traditional wet electrodes [6].

Sweat monitoring is another modality gaining much attention lately in wearable technology, which has application in the detection of early signs of skin dehydration and imbalances in the internal body fluids produced by intensive physical activities or some autonomic dysfunctions, such as hyperhidrosis and diabetes. Laboratory methods for measuring sweat are vast and include Miner’s method [7], “wash-down” techniques [8], Parafilm patches [9] and impedance [10]. From all these methods, only impedance can be performed for extended periods of time outside laboratorial settings. However, impedance involves complex circuitry for *wearables* and it is neither completely immune to movement artifacts nor electrode placement. In this paper, we reduce the complexity in electronics by allowing a single excitation frequency in the lower spectrum (1 kHz) where the conductivity effects dominate within the biological tissues, introducing also a new matrix made of electrodes to cover the tissues under analysis more uniformly. Such strategy avoids the problem related to electrode placement, while providing an area for sweat spreading (and dissipation), slowing down the process of channel saturation. In opposition to the approaches that use water vapor flow measurements [11] and humidity sensors printed on textiles [12], our solution employs a smaller and removable detection matrix, which minimizes the disturbance in the microclimate close to the skin where the sweat glands are located. Sweat also contains electrolytes that can be further analyzed such as pH and lactate for assessment of the athletes’ performance or personal wellbeing [13]. pH is the analyte detected by means of a voltammeter transduction mechanism [14], wherein the concentration of protons in solution increases the voltage potential around an active electrode in reference to a bias level. The active pH electrode can be deposited using different techniques such as screen printing [15,16,17], dip casting, electrochemical deposition [18] and tailored according to the requirements using, in some cases, gold modified hydrogen ionosphere and plasticized PVC or graphene-polyurethane composites [19]. The typically high impedance of the electrode must be matched in the recording electronics by an ultra-low input bias current amplifier in order to reduce measurement errors introduced by the multiplication of the bias current of the amplifier by the electrode’s impedance, masking the potential generated by the protons in solution (around 56 mV/ unit pH). By its turn, lactate is recorded amperometrically by immobilizing lactate enzyme inside a sensing membrane and careful tailoring of the sensing layers to prevent unwanted interferences from other molecules in solution [20]. Lactate is then ionized and flows accordingly to the electric field imposed between a counter and reference electrodes, being detected through a working electrode that transforms this ionic flow into electrical current. Within this study, we deposited pH and lactate sensors over gold-platted electrodes located on a removable ear support, which contains also the electrodes for impedance and ECG. An additional membrane protects the chemical sensors from movement interferences, which constitutes a novelty when compared to the laboratory platforms found in the aforementioned literature, yet porous enough to allow sweat to flow. For the case of intense exercise leading to a faster saturation of the measurement channels, the ear support can easily be detached from the main circuitry board, washed and new sensors deposited again, assuring the reuse of the proposed technology. 

**Figure 1:**
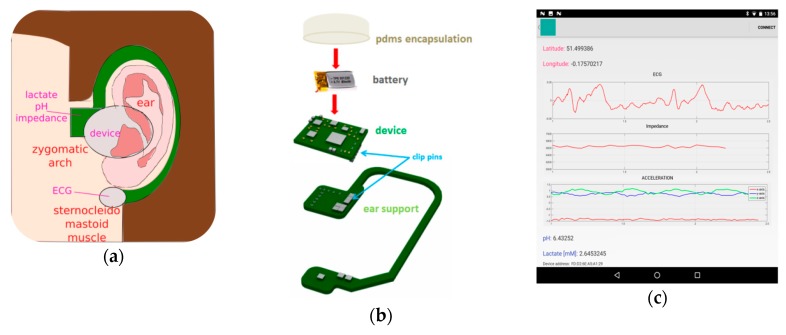

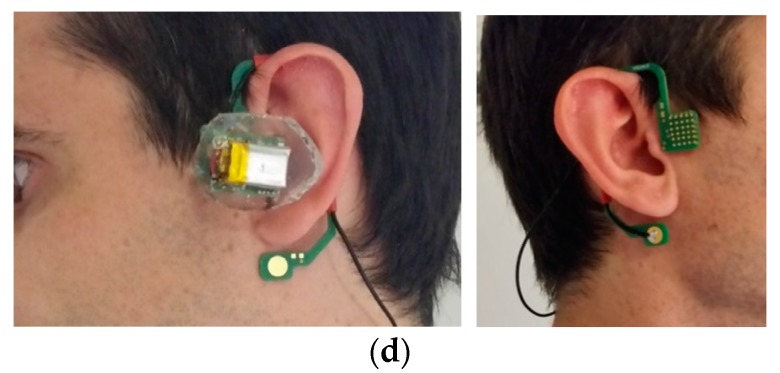
Prototype developed for measurement of cardiovascular, motion and sweat parameters around the ear, with total weight of 50 g: (**a**) Main anatomical features located around the left ear for acquisition of the ECG, impedance and chemical analytes through the skin; (**b**) Prototype assembly, involving device connection to an external battery, followed by PDMS encapsulation for protection of the electronics without compromising BLE transmissions and attachment to the ear support through clip pins; (**c**) Mobile phone’s app developed to receive the BLE signals originated at the device; (**d**) Location of the assembled prototype over the left ear, while the right one holds the ear support only (although flipped) to contact the ECG electrode with the skin.

From all the exposed above and in summary, in this paper we propose an ear-worn device to measure cardiovascular and sweat parameters during exercise with BLE connectivity. Unlike the approach followed by the authors of [21], the proposed device records bipolar ECG signals around the ears, whereas sweat rate is estimated by the impedance method. Recording of pH and lactate levels as well as head motion complements the specifications of the device, especially designed as an unobtrusive earpiece shown in Figure 1. Its location has been selected to facilitate access to the sweat accumulating in the upper part of the cheek (zygomatic arch) in the left side of the head, while the detection of the ECG signal occurs over the sternocleidomastoid muscle, hence avoiding the surrounding (non-conductive) bone areas. Our approach is to integrate multimodal sensing into a single device by making use of discrete *off-the-shelf* components. Another approaches use either large commercial chipsets or *system-on-chip* design into a custom-made smaller chip, which can integrate many functions and sensing capabilities by reconfiguring the basic analogue front-end blocks involved in the measurement of bio-potential, impedance and/or chemical analytes [22,23]. However, this latter approach has several disadvantages including longer design-to-production times and the inability to measure all modalities in simultaneous, besides requiring logical signals and reference clocks to select the desirable configuration, filter tuning and control that adds extra modulation noise originating from external complex digital circuitry. Moreover, multiplexing of input signals to meet the requirements of reconfigurable electronics is never a good option as it degrades the input impedance of the first amplifier on the electronic chain as seen directly by the source of the input signals, which must remain as higher as possible for detection of tiny bio-potential signals and/or pH level, as performed in this study.

## 2. Materials and Methods

**Figure 2:**
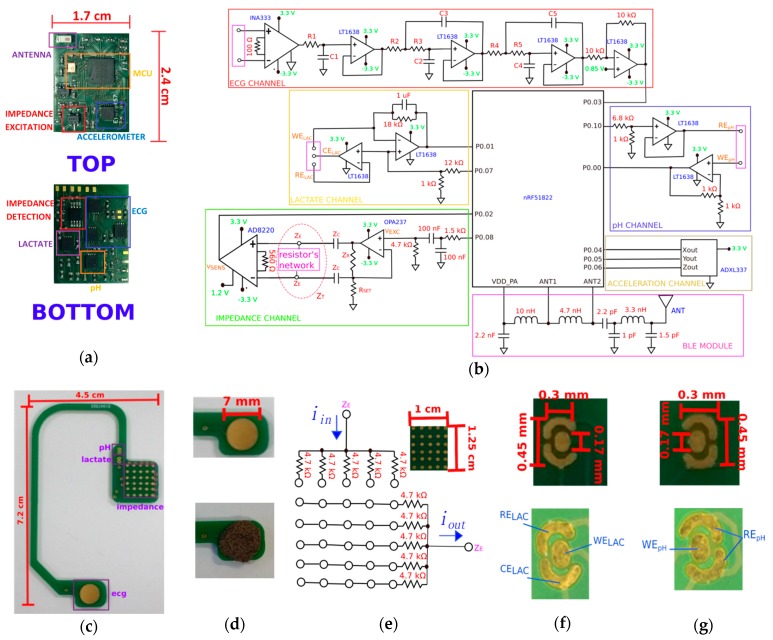
Different parts composing the ear-worn device: (**a**) Main electronic board with components assembled in the top and bottom layers; (**b**) Simplified electronic schematic covering the ECG, impedance, lactate and pH measurement channels; (**c**) Ear support developed to contact the electrodes and the sensors with the tissue around the ears; (**d**) ECG electrode covered with conductive textile; (**e**) Matrix of electrodes for impedance measurements, with equivalent resistors’ network; (**f**) Lactate sensor deposition (WE_LAC_) with RE_LAC_ and CE_LAC_ electrodes, as captured by microscope (VHX-5000, Keyence, Milton Keynes, UK); (**g**) pH sensor deposition (WE_pH_) with single reference electrode (RE_pH_).

This section describes in detail the electronic modules inside the wearable device, namely the ECG, impedance, pH and lactate measurement channels, as depicted in Figure 2a,b, for the layout of the printed circuit board and schematics, respectively. Electrode development for each of the aforementioned modalities is also thoroughly covered in this section. At the end, a description of the mobile phone app developed to receive the wireless signals finalizes the section.

### 2.1. ECG Measurement Channel

For ECG detection, a low noise instrumentation amplifier (INA333 from Texas Instruments, Dallas, TX, USA) was used with high amplification gain (1000 V/V) to differentially amplify the signals around the upper part of the sternocleidomastoid muscle, as depicted in Figure 2b (ECG channel). The requirement for bipolar measurements has led to the use of a shielded wire to reduce noise interferences, routing the signal from the right ear to the left side of the head (device location). Each signal was picked-up by two electrodes made of woven conductive textile (Stretchy silver-plated nylon, Kitronik Ltd, Nottingham, UK), attached to the gold-platted pads on the ear support by silver conductive adhesive epoxy (8331-A, MG Chemicals Ltd, Surrey, BC, Canada), vide Figure 2d. Although these bare-gold pads can themselves pick-up the ECG signal, their flat surface is more prone to lose contact with the skin during motion, as opposed to textiles, which have a more porous and thick structure (larger contact area), yet soft enough not to cause skin bruises by constant rubbing or friction of the electrodes over the skin. After signal amplification, a 5^th^- order lowpass filter with cut-off frequency (**f_cut_**) around 20 Hz was designed with standard operational amplifiers (LT1638, Analog Devices, Norwood, MA, USA) to reduce noise interferences, specially the capacitive coupling originating from the power grid line. Dimensioning of the resistors on the filter to meet the frequency requirement was calculated in accordance to Expression (1) by imposing the capacitors’ values as **C_1_** = 4.7 μF, **C_2_** = 15 nF, **C_3_** = 33 nF, **C_4_** = 8.2 nF and **C_5_** = 100 nF, and selecting the closest nominal values for the resistors as provided by the manufacturer, hence the approximations in the following formulas:

(1) $$R_{1}=\frac{1}{2{\pi f}_{\mathrm{cut}}C_{1}}≃1.8\;k\Omega ,\;{\;R}_{2}=\frac{a_{2}C_{3}-\sqrt{a_{2}^{2}C_{3}^{2}-4C_{2}C_{3}}}{4{\pi f}_{\mathrm{cut}}C_{2}C_{3}}≃190\;k\Omega \;\;\left(a_{2}=1.62\right),\;R_{3}=\frac{a_{2}C_{3}+\sqrt{a_{2}^{2}C_{3}^{2}-4C_{2}C_{3}}}{4{\pi f}_{\mathrm{cut}}C_{2}C_{3}}≃670\;k\Omega ,\;{\;R}_{4}=\frac{a_{3}C_{5}-\sqrt{a_{3}^{2}C_{5}^{2}-4C_{4}C_{3}}}{4{\pi f}_{\mathrm{cut}}C_{4}C_{3}}≃190\;k\Omega \;\;\left(a_{3}=0.62\right),\;R_{5}=\frac{a_{3}C_{5}+\sqrt{a_{3}^{2}C_{5}^{2}-4C_{4}C_{3}}}{4{\pi f}_{\mathrm{cut}}C_{4}C_{3}}≃2\;M\Omega$$

The filter output was then digitalized with 10-bit of resolution by the embedded microcontroller (nRF51822, Nordic Semiconductor, Trondheim, Norway) at rates of 140 SPS for continuous segments of 30 s, interleaved by the measurement of the impedance, pH and lactate signals, as described in the next sections.

### 2.2. Impedance Measurement Channel

For impedance measurement of sweat, the bipolar configuration was employed that uses the same pair of electrodes for current injection and voltage detection. Electrodes were gold-plated not only to assure biocompatibility with the surrounding tissues, but also to lower impedance contact with the skin. A voltage-to-current converter (VI) was adopted in this study to transform a TTL signal generated by the microcontroller (MCU) into a sinewave (V_EXC_) with a frequency of 1 kHz, by low-pass and high-pass signal filtering before VI conversion (OPA237, Texas Instruments, Dallas, TX, USA), as shown in Figure 2b (impedance channel). The total circuit load (Z_L_) present in the feedback loop of the VI converter was determined beforehand in order to accommodate for possible impedance discrepancies generated over the interfaces device-electrodes and electrodes-tissue as:

(2) $$\frac{1}{Z_{L}}=\frac{1}{Z_{R}}+\frac{1}{2Z_{C}+2Z_{E}+Z_{T}}\Leftrightarrow Z_{L}=\frac{2Z_{C}+2Z_{E}+Z_{T}}{\frac{2Z_{C}}{Z_{R}}+\frac{2Z_{E}}{Z_{R}}+1}$$

where **Z_R_** is the impedance of the closed-loop resistor (560 kΩ), **Z_C_** the impedance of the DC-blocking capacitors, **Z_E_** the impedance of the electrodes and **Z_T_** the impedance of the ear segment. By forcing **Z_R_** >> **Z_T_**, **Z_E_** → 0 and **Z_C_** → 0 for 1 kHz excitation, last expression yields **Z_L_** ≃ **Z_T_**. For a unitary voltage signal, the current-setting resistor (**R_SET_**) was chosen to 560 kΩ in order to impose a current limitation around 2 μA to the living tissue. Within this condition, a lower value of impedance must be produced by **Z_T_** to prevent VI malfunctioning. By its turn, DC-blocking capacitors were added to the circuitry to prevent direct currents to flow into the head segment, thus avoiding electrolysis and the effects of permanent migration of free ions within the medium that occur during long-term recordings [24]. Limitation for AC current stimulation through the body is set to a magnitude of 100 μA, whereas skin impedances for humans are reported within the low kilo Ω range (lower spectrum), though a precise value is undefined as it depends on factors such as skin ageing, hydration level and tissue composition [24]. Regarding these two factors, we decided to produce an even smaller current so that the voltage drop produced by the skin could still be accommodated by the VI converter (≤power supply level) in the eventuality of higher impedances present.

By its turn, for voltage detection, an instrumentation amplifier (AD8220, Analog Devices, Norwood, MA, USA, gain of 90 V/V) was selected to sense the signal developed across the electrodes (**V_SENS_**). The output of the amplifier is then digitalized at rates of 8 kSPS, yielding a total of 80 data points (8 points per period) employed in the estimation of the magnitude of **Z_T_** by the Discrete Fourier Transform (DFT) as:

(3) $$\mathrm{Real}\left\{X_{50}\right\}=\sum_{n=0}^{N}x_{n}\cos\left(-j2\pi \times 50\frac{n}{N}\right),\;\mathrm{Imag}\left\{X_{50}\right\}=\sum_{n=0}^{N}x_{n}\sin\left(-j2\pi \times 50\frac{n}{N}\right)\;$$

where **x_n_** is the 80-point sample vector, **n** the sample index, **N** the total number of samples and **X_50_** the 1 kHz component. Conversion into amplitude is performed already inside the mobile phone’s app by Expression (4) as it involves mathematical operations hard to implement inside the MCU:

(4) $$\left|Z_{T}\right|=R_{\mathrm{SET}}\frac{\sqrt{\mathrm{Real}{\left\{X_{50}^{\mathrm{SENS}}\right\}}^{2}+\mathrm{Imag}{\left\{X_{50}^{\mathrm{SENS}}\right\}}^{2}}}{\sqrt{\mathrm{Real}{\left\{X_{50}^{\mathrm{EXC}}\right\}}^{2}+\mathrm{Imag}{\left\{X_{50}^{\mathrm{EXC}}\right\}}^{2}}}$$

An additional resistor network or matrix consisting of 6 × 5 millimeter-sized gold-plated electrodes was developed to detect more accurately the propagation of sweat around the left ear, as depicted in Figure 2e. This matrix of electrodes was projected to drive the imposed current through the area as soon as sweat enters in contact with the electrodes, lowering network’s impedance. 

### 2.3. pH and Lactate Measurement Channels

The pH measurement channel was developed by chemical deposition of a specially-designed pH sensor (WE_pH_) over the gold-platted electrodes in the ear support connected to the pH acquisition channel. The electronics for this channel employed a non-inverting amplifier (LT1638, Analog Devices, Norwood, MA, USA, gain of 2V/V) with reference voltage (RE_pH_) set to 0.42 V by means of a voltage follower, as shown in Figure 2b (pH channel). By its turn, the lactate channel incorporated a specialized lactate sensor (WE_LAC_) connected to a transimpedance amplifier (current amplification of 18000x) with counter (CE_LAC_) and reference (RE_LAC_) electrodes set to a potential of 0.25 V, vide Figure 2b (lactate channel). In terms of the fabrication process, the pH sensor was electrochemically deposited on an Ir wire forming IrOx metal-metal oxide pH sensor. The surface of the sensor was first electrochemically cleaned before membrane deposition. An intermediate layer of PEDOT was deposited to serve as a stabilising layer. Iridium oxide pH electrodes were then achieved through electrochemical oxidation during potential cycling between 0.2 V and 0.8 V. The iridium oxide nanoparticle dispersion was made using 3.0 mM aqueous K_2_IrCl_6_ solution adjusted to pH 10 with 7% wt of aquous NaOH. The resulting solution was cooled to room temperature and then adjusted to pH 2 by rapidly adding 1 M HNO_3_ and stirred continuously for 40 minutes. For lactate, a dip coating procedure was followed for deposition of the sensing membrane. The electropolymerisation method for phenol red was used as an internal medium layer for the WE_LAC_, by potential cycling between +0.35 V and +1 V. The drop-casting solution contained enzyme 60 mg/mL glucose oxidase (GOx) from *Aspergillus niger* (Sigma Aldrich, St. Louis, MO, USA) and 30 mg/mL bovine serum albumin in 0.01 M PBS. After enzyme immobilization, the sensor was additionally coated with a polyurethane film with 4 different concentrations (2 to 5%) in order to extend its dynamic range. The developed sensors shown in Figure 2f,g were additionally covered with a protective membrane made of biocompatible polyurethane to prevent easy detachment from the ear support, yet porous enough for sweat to flow through and analytes to be detected by the sensors below. 

### 2.4. Mobile Phone’s App and Power Consumption

A graphical user interface was created in Android Studio to receive the data packets sent by the device via BLE, as shown in Figure 1c. The microcontroller inside device’s electronics was selected in part because it contained a dedicated low-power 2.4 GHz transceiver for BLE communication, which was easy to implement and tune in hardware by using some external resistors and capacitors, creating an RF matching circuitry (Pi-network) connected to a 50 Ω single-ended antenna (2450AT18B100, Johanson Technology, Camarillo, CA, USA), see Figure 2b (BLE module). Transmissions were scheduled to occur every 100 miliseconds with data containing the samples acquired from each channel plus the information collected by a 3-axis accelerometer (ADXL337, Analog Devices, Norwood, MA, USA) with resolution of 0.01 g and range of ±1.5 g, depicted in Figure 2b (acceleration channel). The data payload was set to 20 bytes per BLE packet, translated into a data communication rate of 1.6 kbps, with transmissions lasting for 10 ms and peak current consumption around 15 mA. ECG samples are streamed continuously in 30 s segments, followed by 5 s segments containing, alternatively, the impedance, lactate and pH values. ECG data occupies 18 bytes (14 samples × 10-bit) inside the packet, whereas the coefficients for impedance occupy 8 bytes, lactate/pH values are transmitted within 10 bytes (8 samples with 10-bit resolution each) and the accelerometer information is compressed to 2 bytes only (5-bit resolution for each axis). An extra byte included in the BLE packet distinguishes between different modalities at the receptor’s side.

In terms of power consumption, the device presents two different profiles that translate the type of operation being performed by the MCU during its active cycle: data acquisition and data transmission. For the former, a current consumption of 15 mA was measured by a handheld multimeter (72-7730A, Tenma, Leeds, UK), while the latter consumed 25 mA (15 mA for communication plus an additional current of 10 mA drawn by the rest of the electronic components). Since the temporal extension of these operations is different, with acquisition occyping 90% of the cycle, the total power consumption is:

(5) $$t_{\mathrm{ACQ}}\times I_{\mathrm{ACQ}}+t_{\mathrm{TRANS}}\times I_{\mathrm{TRANS}}=16\;\mathrm{mAh}$$

where **t_ACQ_** and **t_TRANS_** are the duration of the acquisition and transmission periods, respectively, whereas, **I_ACQ_** and **I_TRANS_** represent the current consumption for the two operations. By employing an 80 mAh rechargeable battery (501220, TPE New Energy, Shenzhen, China), the device can operate continuously for periods of 5 h, enough to cover the physical activities performed in most of the sport modalities.

Finally, inside the graphical user interface, the ECG samples are further processed by means of a 10th-order lowpass filter (**f_cut_** = 10 Hz), whose finite impulse response for the **k** data sample is given by Expression (5). Filter coeficients were obtained by the Filter Design & Analysis Toolbox from Matlab (Mathworks Inc., Natick, MA, USA) as part of a Gaussian window-type FIR with α set to 2.5:

(6) $$\begin{array}{ll} y\left[k\right]=0.0855x & \left[k-1\right]+0.0886x\left[k-2\right]+0.0913x\left[k-3\right]+0.0932x\left[k-4\right]+0.0943x\left[k-5\right]\\ & +0.0947x\left[k-6\right]+0.0943x\left[k-7\right]+0.0932x\left[k-8\right]+0.0913x\left[k-9\right]\\ & +0.0886x\left[k-10\right]+0.0856x\left[k-11\right] \end{array}$$

## 3. Results

This section is divided into the results obtained during the testing of the different channels of the device in laboratory, followed by experimentation with a real exercise. Whenever relevant, simulations of the propagation of the electric potential generated either by the heart or impedance channel will be also presented, as a complement to the results obtained by the device. Derivation of the mathematical formulation involved in these numerical simulations is described in Appendix A. 

### 3.1. ECG Channel Performance in Laboratory

**Figure 3:**
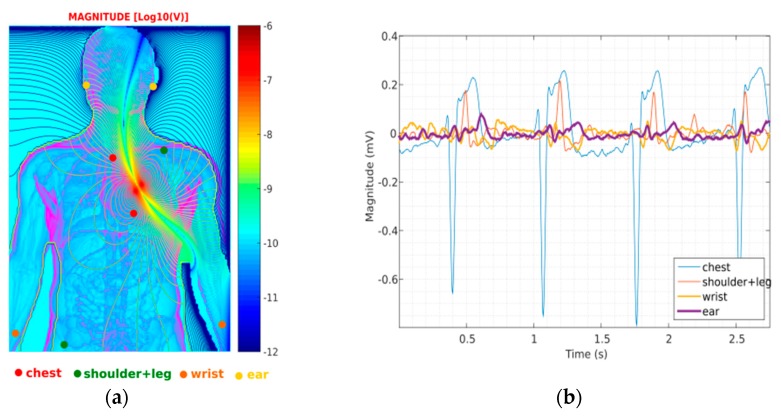

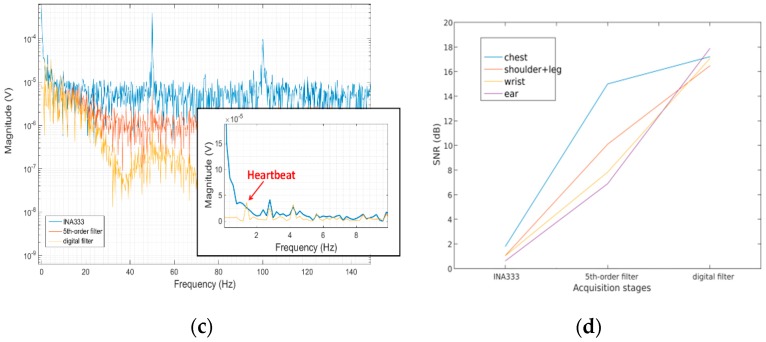
Testing of the ECG channel in different locations of the recording electrodes over the body: (**a**) ECG lead locations – chest, shoulder/leg, wrists and ears - superimposed over a depolarization map of the electric field generated by the heart; (**b**) ECG traces originating in the aforementioned body locations; (**c**) Spectrum for the ECG signal recorded around the ears and acquired at different stages in the electronic circuitry: amplification (INA333), analogue filter (5th-order) and digital filter, highlighting the noise reduction along the different stages and detail of the components closer to DC more related to ECG events; (**d**) Evolution of the SNR along the different electronic stages.

The single channel ECG was recorded in different electrode positions over the body as depicted in Figure 3a, namely, chest (sternum + 5th intercostal space), left shoulder/right leg, wrists and ears, hence providing a means of comparison between ECGs recorded at different electrode positions.The person remained in a seated position for the entire duration of the experiments, without performing any activity. These ECG signals originating at different locations were not acquired at the same time, but were synchronized *a posteriori* by their QRS complexes, as shown in Figure 3b. Voltage signals from different electronic stages within the device were also measured by an oscilloscope (MSO-X 3054A, 50 kSPS, Agilent Technologies, Santa Clara, CA, USA) for further analysis in the frequency domain, *vide* Figure 3c. Finally, the signal-to-noise ratio (SNR) of these signals was calculated in order to evaluate their quality (Figure 3d). In the computational domain, each signal was first low-pass filtered with cut-off frequency at 30 Hz (10th - order) to yield a desired noise-free signal, whereas the noise model was derived from the high-pass filtered version of the same signal with equal cut-off frequency and filter order. SNR values obtained this way correspond, therefore, to the ratio between the squared magnitudes of the 30 Hz ECG signal by its noise model. 

### 3.2. Impedance Channel Performance in Laboratory

**Figure 4:**
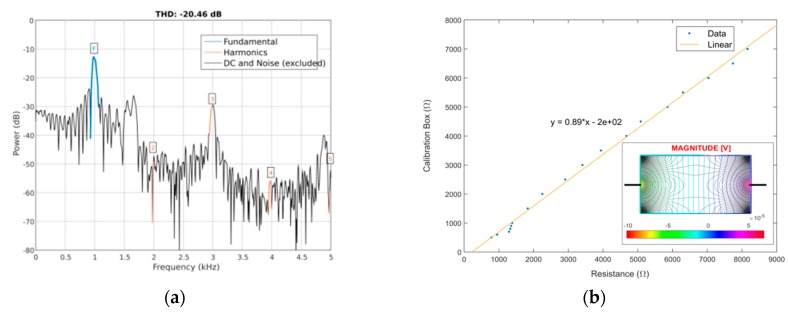

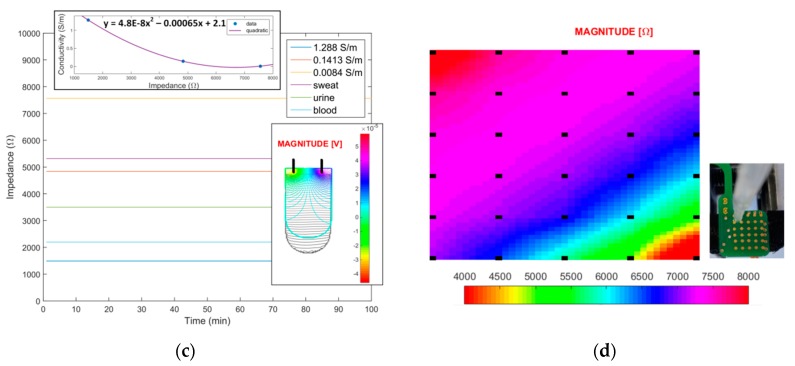
Characterization curves obtained for the impedance channel: (**a**) Power spectrum of the excitation signal, with separation between fundamental, harmonics and noise peaks; (**b**) Calibration of the channel by means of a calibration box, with a model for the propagation of the isocurrent (gray) and isopotential (color) lines presented in the inlet (σ_resistor_ = 1 S/m); (**c**) Impedance value obtained for conductivity solutions and body fluids, with respective calibration curve and model for the propagation of the isocurrent and isopotential lines inside the centrifuge tube (σ_tube_ = 0.66 S/m); (**d**) Impedance distribution map obtained while pipetting a 0.1413 S/m solution along the resistors’ network, from the electrodes located on the top left corner of the matrix to the bottom right ones.

The excitation signal produced by the device was acquired by the oscilloscope (rate of 100 kSPS) before entering VI conversion for the calculation of the total harmonic distortion value (THD), defined as the ratio between the first five harmonics of the signal and the fundamental, as shown in Figure 4a. Then, the entire impedance channel was tested with a calibrated resistor’s box (type 008-B, Cropico, Peterlee, UK) to evaluate the response of the DFT algorithm to standardized resistance values. In this scenario, the current-injecting and voltage-sensing electrodes were connected to the terminal inputs of the box, thereby forcing all the current to flow inside the resistor. The graphic obtained by this procedure is presented in Figure 4b with respective linear fit. After calibration, the device was further tested with conductivity solutions (HI7030/31/33, Hanna Instruments, Woonsocket, RI, USA) as well as some body fluids. The samples were pipetted to a 0.6 mL centrifuge tube at room temperature and covered with a specially designed cap containing a 1 × 2 pin header connector (2211S-02G, 2.54 mm pitch, Multicomp, Leeds, UK) working as electrode and immersed to a volume of 0.5 mL inside the liquid (Figure 3c). Finally, the resistors’ network in the ear support was experimented by pipetting a 0.1413 S/m solution over the area covered by the matrix of electrodes and allowing the droplet to expand until all the electrodes were immersed (volume of 0.3 mL), while recording the respective impedance signal (Figure 4d). 

### 3.3. pH and Lactate Channels Performance in Laboratory

**Figure 5:**
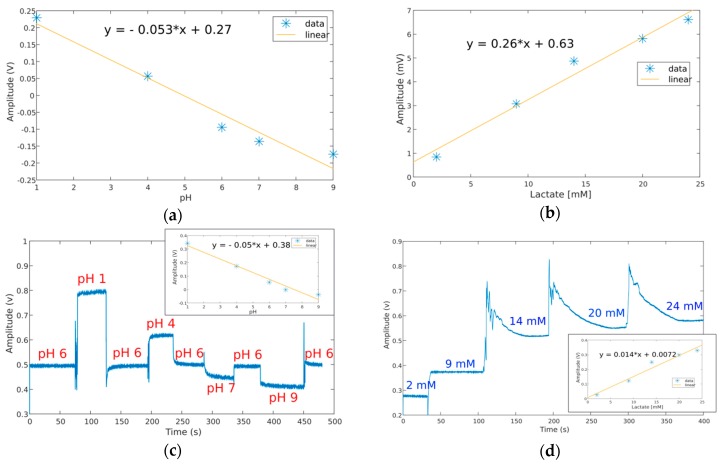
Characterization of the chemical sensors and measurement channels: (**a**) Calibration curve obtained for the pH sensor using a commercial electrochemical workstation; (**b**) Calibration curve obtained for the lactate sensor using the same workstation; (**c**) Temporal recording obtained by the pH measurement channel of the device and respective calibration curve for the tested solutions; (**d**) Temporal recordings and respective calibration curve for the lactate measurement channel while testing with different concentration solutions for the analyte.

The chemical sensors were tested after production with commercial solutions for pH with levels of 1, 4, 6, 7 and 9, followed by lactate with concentrations of 2, 9, 14, 20 and 24 mM. The calibration curves in terms of the voltage level *per* unit of the electrolyte are shown in Figure 5a,b, for pH and lactate, respectively, as obtained by a commercial electrochemical workstation (CHI600E, CH Instruments, Austin, TX, USA). Then, the same solutions were tried after attaching clean sensors to the ear support and connecting to the device. A droplet from each solution was pipetted over the respective electrode area with the signals obtained depicted in Figure 5c,d. 

### 3.4. Testing of the Device in Real Exercise

**Figure 6:**
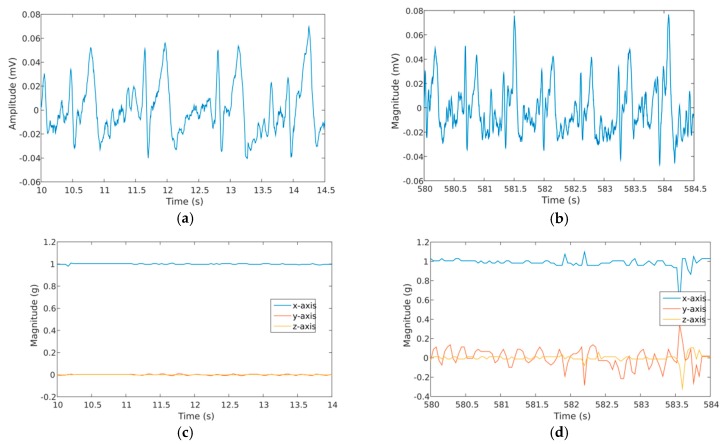
Temporal recordings obtained for one subject performing the indoors cycling exercise and wearing the proposed device: (**a**) ECG signal recorded at the beginning of the trial; (**b**) ECG obtained at the middle of the physical activity; (**c**) Acceleration registered at the beginning of the trial; (**d**) Acceleration obtained at the middle of the physical activity.

**Figure 7:**
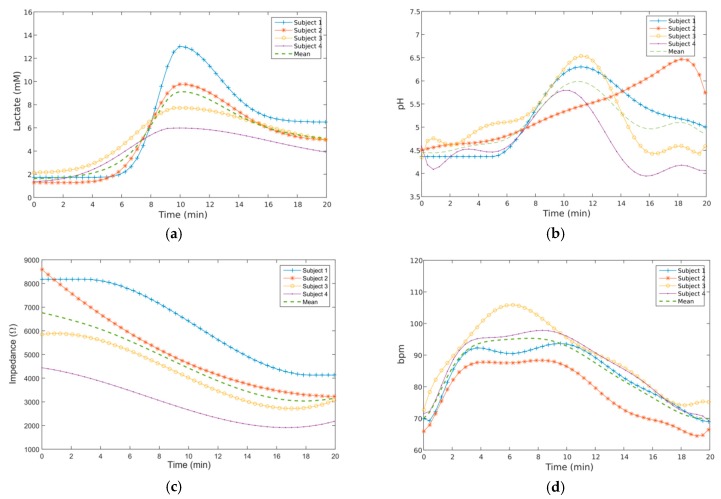
Variation of the physiological parameters during exercise (first 10 min) followed by a recovery period, as obtained for all subjects: (**a**) Lactate; (**b**) pH; (**c**) Impedance; (**d**) Heart rate.

With the device fully tested and calibrated, indoors cycling exercise was performed by four volunteers using specialized equipment (Trainer 2013, Wattbike, Nottingham, UK). The trial lasted for a period of 20 min, divided into a 10 min segment dedicated to exercise at a constant pace, followed by a 10 min period for recovery. An additional adhesive film (model 9780, 3M, Maplewood, MN, USA) was placed over the ear support and behind the ear to increase its fixation to the skin. BLE signals were acquired by a mobile phone (SM-G900F, Samsung, Seoul, Korea) running the developed app and located near the equipment. Ethical approval for this human trial was obtained from Imperial College London (ICREC 18IC4816) with each subject signing a consent form after being fully informed about the experiment. Figure 6 exhibits ECG and acceleration traces obtained at the beginning and middle of the exercise for the same volunteer, whereas Figure 7 shows the physiological signals measured for all subjects. 

These signals were digitally processed *offline* by means of a 5-point moving average filter to eliminate some signal spurious from the recordings and smooth-out transitions between levels for better visualization. Heart rate measurement was performed by manually counting the number of QRS complexes as no algorithm was developed inside the graphical user interface to measure HR automatically. 

## 4. Discussion

The results obtained for the ECG channel have shown signals with higher SNR value for the chest lead, followed by the shoulder/leg, wrist and ear region, after amplification. The SNR levels obtained after amplification (INA333) are still relative small, which is reflected by the presence of a large amount of noise in the spectrum, especially from the 50 Hz power-grid (Figure 3c). Subsequent analogue and digital filtering has elevated the SNR to allow digitalization of the ECGs with moderate QRS definition, enough to estimate the heart beat even at the level of the ears, but with complete absence of the P-wave as experienced already by the authors of [21]. The measuring equipment employed to calculate SNR in this study also introduced additional noise and measurement errors to the values, though it was the only method available to access the target pins directly over the electronics. Moreover, defining the frequency components that belong exclusively to ECG events from those arising from other sources (including noise) is not a trivial process, if commercial and calibrated ECG recording equipment is not available for comparison. In any case, from Figure 3b, one can see that the signals acquired in the chest have the highest amplitudes and ears the lowest of all. This is further corroborated by the simulation model in Figure 3a, where the electric dipole propagates with higher intensity along the chest area, followed by the propagation into the limbs and finally to the head segment. 

Regarding impedance, the excitation signal generated by the device yielded a THD value of −20.46 dB (Figure 4a). This relative low value for THD is a consequence of filtering the square signal produced by the MCU with 1st-order filters only. This drawback was compensated by performing impedance calibrations using resistors and solutions with stable resistance values in the frequency domain, instead of relying directly on the result given by Equation (4). As the frequency of excitation is relatively slow (1 kHz), the conductivity effects on the materials being tested prevail over permittivity and the dispersion effects. Conductivity effects are more frequency-independent and their influence can be understood by applying a simplified version of the Ohm’s law. Within this regard, the loss in impedance magnitude is proportional to the decay induced by the influence of signal harmonics over the fundamental, whose real impedance value for materials and tissues can then be recovered by calibration. From the calibration graph in Figure 4b, it can be seen that the device responds to a range between 1 kΩ and 8 kΩ, with a step resolution close to 10 Ω, a value obtained by considering the resolution of the acquisition channel (10-bit) and the interval of impedance values measured. The 10 Ω resistor produces a voltage of 20 μV, which is already above the noise level of the differential amplifier employed in the acquisition channel (14 nV/√Hz) within a 1 kHz bandwidth (14 μV). By its turn, measurement of the impedance value for solutions with different conductivities has allowed to obtain a fitting curve employed latter to detect the true conductivity level of some body fluids, independently of the geometry of the electrodes and sample container (Figure 4c). Conductivity levels measured by this method were 0.9 S/m for blood, 0.08 S/m for sweat and 0.4 S/m for urine. Compared with literature reference (Table A1, Appendix A), these values are higher by almost a value of 0.2 S/m (blood and urine only), though the experimental conditions were not the same between the current experiment and literature. For the simulation model in Figure 4b, the isocurrent lines for the resistor cross its entire extension, yielding potential lines symmetrically distributed between the recording electrodes, whereas in Figure 4c, some of the current lines escape from the top of the solution and their contribution to the voltage level detected is not accounted for, a fact that might explain the aforementioned discrepancies in conductivity. 

For the chemical sensors, a sensitivity level of 53 mV/pH was measured for the fabricated pH sensor (Figure 5a), which declines to a level of 50 mV/pH with the electronic device (Figure 5c). The bias current at the input terminal of the non-inverting amplifier can be responsible for this decline as well as the circuit board traces connecting the pH sensor to the amplifier, introducing further measurement errors. Nevertheless, the device with pH sensor attached was able to distinguish the pH from different solutions by producing proportional voltage levels. By its turn, for lactate, an increasing voltage level was obtained with the concentration of the analyte in solution for both the electrochemical workstation (Figure 5b) and proposed device (Figure 5d). For the latter, the sensibility is higher for the lower concentrations of lactate (<20 mM) reflected in the higher voltage difference between signal baselines in Figure 5d. The complete lactate measurement channel saturates for concentrations close to 20 mM as a consequence of the current amplification level set by device electronics. In the present conditions and attending to the fitting curve obtained, the voltage difference between tested solution (≃20 mM) is 0.25 V, a value that divided by the resistor employed in the transimpedance amplifier (18,000 kΩ) yields a detectable current level of 14 μA or a standardized sensitivity level of 0.8 μA/mM for the sensor. 

Finally, in what concerns the physical exercise, the parameters that experience faster changes are heart rate and impedance (Figure 7), with pH and lactate experiencing an abrupt rise at the middle of the indoors cycling event. Moreover, in the 10 min period allowed for recovery, these parameters do not return to their initial levels, in opposition to ECG, with all subjects going back to pre-exercise HR. By its turn, the impedance level never recovers completely from the decline proportionated by exercise, suggesting that some sort of micro fluidic channel must be included within the matrix to remove more efficiently the sweat during intensive physical activities or replace the ear support by a freshly new one in order to continue monitoring. At the end, the mean variation of the physiological parameters measured among all subjects during the trail are pH = 5.10 ± 1.54, LAC = 5.26 ± 7.50 mM, IMP = 4.59 ± 3.73 kΩ and HR = 85 ± 25. 

## 5. Conclusions

An ear-worn device for cardiovascular and sweat monitoring has been experimentally tested under both laboratory and real exercise conditions. The need to confine the electronics into a small low-powered device has led to some simplifications in terms of circuitry implementation, namely the absence of higher-order filters to increase the THD of the excitation signal (impedance) and cascade amplifiers for the pH and lactate channels. Measurement of the phase for impedance was also not performed in this study as it remains unclear its influence in the course of the biological processes occurring inside tissues. The limitation on the acquisition rate of the internal ADC of the microcontroller (<10 kSPS) prevented the use of higher frequencies for impedance stimulation as well. By its turn, the absence of a driven right leg circuit for ECG had repercussions in the lower SNR values obtained in the first stage of signal amplification. With more robust electronics, we believe that it is still possible to detect all features of the ECG around the ears if higher ADC resolutions are employed (16-bit as commonly employed by front-end components in medical equipment) together with better shielding of device electronics and body segment.

To the best knowledge of the authors, this is the first study that combines the measurement of the cardiovascular performance with sweat parameters that has been fully integrated into a single ear-worn device. Control of the individual effort in physical activities is of paramount importance in sports in order to avoid injuries due to unadjusted intensive strains, a situation that the device can help prevent if overstrain is detected on the levels of the measured signals in real-time.

Finally, as future work, miniaturization of the device in terms of electronics can still be achieved externally by *system-on-chip* integration for every sensing modality independently or, internally, by means of an implantable device. In this latter scenario, bipolar ECG recordings are no longer feasible and HR estimation must be performed by means of optical measurements, such as pulse plethysmography over the earlobe. A flexible circuit board can also be designed for the ear support to adjust more naturally to the contour of the head and ears, though the capacitive substrates employed in the process may alter the impedance measurements. In the chemistry point-of-view, other analytes can also be detected in sweat by incorporation of new sensors with specialized membranes tailored to detect them. In this scenario, the specificity of each sensor must be individually evaluated against all the chemical species present in the medium, from each lactate is one of the most predominant analytes expelled during exercise. 

## Appendix A

The Finite Integration Technique (FIT) was used to create a simulation domain to evaluate the propagation of the electric potential generated by the heart (endogenous source) or external source (impedance). FIT has long been used to implement Maxwell’s equations on electromagnetism into a discrete formulation suitable for computers, while dividing the domain into a limited number of regular cell-elements [25,26]. In this study, the domain was divided into N = 40000 rectangular elements. The calculation of the electric potential inside the domain has followed the principles dictated by the Electrical Impedance Tomography (EIT) by knowing, in advance, the distribution of the conductivity (**σ**) of body tissues within the human body (coronal slice) and **σ** for the materials inside the resistor’s model or centrifuge tube, in accordance to Table A1. Then, the equations for EIT were implemented with the same mathematical simplifications as performed in [27,28], by assuming an electrostatic condition for Ampere’s law and the irrotational nature of the electric field in the harmonic regime of stimulation, thus obtaining:

(A1) $$\nabla .\sigma \nabla_{\Phi }=0$$

where **∇.** is the divergence operator and **∇_Φ_** the gradient of the electric potential **Φ**. In the elements defining the electric stimulus, conditions for neutral electric charges were imposed, in the form of source and sink points, where the electric current could flow in and out, as given by Equation (A2):

(A2) $$\Phi^{in}=-\Phi^{\mathrm{out}}$$

The Maxwell-Grid equations just presented were implemented computationally as a system of the form **Au** = **b**, where **A** ∈ *R^NxN^* represents the product of the topological operators with the conductivity map, **b** ∈ *R^Nx1^* an all-zero column vector except in the pair of elements defining the stimulus (electrodes) and **u** ∈ *R^Nx1^* the solution of the system in terms of **Φ**. Inversion of the system **u** = **A^−1^b** was achieved without pre-conditioning the highly sparse matrix **A**, with the system converging to a solution within a couple of seconds of processing, this using an Intel Core i7-4770 (3.4 GHz) processor with 16 GB of system RAM at the disposal of the numerical routine.

**Table A1:** Conductivity values for some biological tissues and fluids at 1 KHz [24].

Tissue	σ (S/m)	Fluid	σ (S/m)
Skin	0.001	Blood	0.7
Bone	0.02	Urine	0.2
Heart	0.1	Fat	0.03
Lung	0.03	Air^1^	1 × 10^−6^
Muscle	0.3		

^1^ The conductivity of the air is null but, in order to prevent numerical instabilities, a small value was allocated.
